# A Library of Potential Nanoparticle Contrast Agents for X-Ray Fluorescence Tomography Bioimaging

**DOI:** 10.1155/2018/8174820

**Published:** 2018-12-27

**Authors:** Yuyang Li, Kian Shaker, Jakob C. Larsson, Carmen Vogt, Hans M. Hertz, Muhammet S. Toprak

**Affiliations:** Department of Applied Physics, Biomedical and X-ray Physics, KTH Royal Institute of Technology/Albanova, SE 106 91 Stockholm, Sweden

## Abstract

Nanoparticles (NPs) have been used as contrast agents for several bioimaging modalities. X-ray fluorescence (XRF) tomography can provide sensitive and quantitative 3D detection of NPs. With spectrally matched NPs as contrast agents, we demonstrated earlier in a laboratory system that XRF tomography could achieve high-spatial-resolution tumor imaging in mice. Here, we present the synthesis, characterization, and evaluation of a library of NPs containing Y, Zr, Nb, Rh, and Ru that have spectrally matched K-shell absorption for the laboratory scale X-ray source. The K-shell emissions of these NPs are spectrally well separated from the X-ray probe and the Compton background, making them suitable for the lab-scale XRF tomography system. Their potential as XRF contrast agents is demonstrated successfully in a small-animal equivalent phantom, confirming the simulation results. The diversity in the NP composition provides a flexible platform for a better design and biological optimization of XRF tomography nanoprobes.

## 1. Introduction

Nanostructured materials have reached a central role in nanomedicine, in the search of new diagnostic and therapeutic agents. One of their most attractive features is the possibility of influencing their biodistribution by surface decoration with targeting agents. Nanoparticles (NPs) can carry a high payload of contrast-generating material compared with small molecules, and they have a long circulation time. NPs are also used as contrast agents in bioimaging for a variety of microscopy and medical imaging methods. Colloidal gold NPs are applied in electron microscopy and semiconductor quantum dots in visible fluorescence microscopy. Clinically, imaging with NPs has been limited to FDA-approved magnetic iron oxide NPs in MRI [1, 2]. In small-animal imaging applications, however, targeted NPs are investigated as contrast agents in many modalities. NPs based on gold and other materials have been developed as alternatives to conventional contrast media for classical absorption-based X-ray imaging [3–6]. X-ray computed tomography (CT) is a prevalent diagnostic tool, which is based on the mechanism that high-density materials or high atomic number elements tend to absorb more X-rays, and high-Z NPs are therefore capable of increasing the contrast of tissues and organs where they are present. NP contrast agents designed for CT with various elements such as bismuth [4], tantalum [7], platinum [8], ytterbium [9], yttrium [10], gadolinium [11, 12], tungsten [13], and others have also been reported. Furthermore, CT detection of actively targeted gold NPs against lymph nodes [14] or breast tumors [3] has been demonstrated in mice.

X-ray fluorescence (XRF) is a well-established nondestructive quantitative elemental analysis technique that has the ability to accurately probe most heavy elements by detecting the secondary X-rays emitted from the objects irradiated with a high-energy primary X-ray beam. This methodology was first used in medicine for elemental analysis in animal bones and a variety of biological tissues [15–17]. In recent years, XRF has been adopted to in vivo studies for quantification of lead [18], iron [19], and iodine [20]. In the last decade, X-ray fluorescence (XRF) tomography was developed as an imaging technique based on XRF quantitative analysis using monochromatic synchrotron X-rays for elemental analysis of small samples [21–23]. It has been suggested that a benchtop XRF tomography system can be developed and used for the determination of the spatial distribution and concentration of NPs within small animals (i.e., biodistribution) during preclinical studies [24–27].

We recently reported on a laboratory scale XRF tomography system [26] with spectrally matched molybdenum (Mo)-based NPs as the contrast agent, enabling high-spatial-resolution tumor imaging in mice. The X-ray source has a characteristic 24 keV line emission well matched by the K-absorption edge of Mo (20.0 keV, K*α* XRF at 17.4 keV), showing promising potential for in vivo small-animal imaging [28]. To further develop the system based on spectrally matched NPs, it is important to reach a higher local concentration of NPs either via increased injected concentration or targeting schemes. XRF excitation energies of commonly used CT contrast agents are way beyond those of our laboratory system. Consequently, a search for potential XRF contrast agents for this system (with 24 keV line emission) is necessary, which will also allow a more flexible chemical platform for the biological optimization of the NPs.

In order to identify alternative contrast agents, we have screened the K-shell XRF excitation and emission energies of various elements neighboring Mo. We have identified yttrium (Y), zirconium (Zr), niobium (Nb), ruthenium (Ru), and rhodium (Rh) as potential XRF contrasting elements, whose K-shell absorption spectrally match the source energy of our system. Here, we present on the synthesis and characterization of a library of NPs, based on Y, Zr, Nb, Ru, and Rh elements. The potential use of these NPs as XRF contrast agents is demonstrated successfully in a small-animal equivalent phantom, with Mo-based NPs included for comparison, confirming the theoretical modeling results.

## 2. Materials and Methods

### 2.1. Materials

Yttrium nitrate hexahydrate (Y(NO_3_)_3_·6H_2_O, 99.98%, Sigma Aldrich), Ammonium niobate oxalate hydrate (NH_4_NbO(C_2_O_4_)_2_·xH_2_O, 99.99%, Sigma Aldrich), Zirconium oxychloride (ZrOCl_2_·xH_2_O, 99.9%, Alfa Aesar), Ruthenium chloride hydrate (RuCl_3_·xH_2_O, Ru 40–49%, Sigma Aldrich), Rhodium chloride hydrate (RhCl_3_·xH_2_O, Rh 38∼40%, Sigma Aldrich), Urea (CH_4_N_2_O, ≥99%, Sigma Aldrich), Ethylene glycol (HOCH_2_CH_2_OH, 99%, Sigma Aldrich), and Polyvinyl pyrrolidone -PVP ((C_6_H_9_NO)_*n*_, MW = 55 kDa, Sigma Aldrich). All reagents are used as received, without further purification.

### 2.2. Synthesis of NPs

As the final compositions of the particles synthesized in this work are different, their synthesis methods are distinct. Y-, Zr-, and Nb-based NPs are synthesized using a hydrothermal method while Rh and Ru NPs are synthesized by a polyol method.

#### 2.2.1. Y-, Zr-, and Nb-Based Nanoparticles

Hydrothermal synthesis has been used for the synthesis of ceramic NPs of Y, Zr, and Nb, adapted from a method reported earlier [29, 30]. Specifically, 0.03 M Y^3+^, 0.3 M Zr^4+^, and 0.03 M Nb^5+^ solutions were prepared in the presence of 0.5 M urea by dissolving Y(NO_3_)_3_·6H_2_O, ZrOCl_2_·xH_2_O and NH_4_NbO(C_2_O_4_)_2_·xH_2_O in 40 mL of DI water. After few minutes of vigorous stirring, the transparent solution was transferred into a 50 mL stainless-steel Teflon-lined autoclave. Hydrothermal synthesis was performed in an oven at 180°C for 20 h. The precipitates formed were collected after cooling the autoclave to room temperature and then washed with distilled water and ethanol by centrifugation and redispersion cycles several times.

#### 2.2.2. Ru- and Rh-Based Nanoparticles

For the synthesis of Rh and Ru NPs, a polyol synthesis route was adapted, details of which are reported elsewhere [31]. In a typical synthesis, Rh or Ru precursors (0.1 mmol by atom) and a PVP stabilizer (2 mmol by repeating unit) were premixed in 10 mL ethylene glycol using an ultrasonic bath. To assure the complete dissolution of the precursors, the solution was heated up to 65°C. Thereafter, the solution was transferred into a 50 mL three-neck flask fitted with a condenser, and the target temperature was reached and maintained using a digital controller with a glass-coated thermocouple and a heating mantle. The solutions were first heated to 90°C for particle nucleation, visualized by a darkening of the solution. After holding at 90°C for 15 min, the solutions were heated and maintained for 1.5 h to a focusing temperature of 160°C for Ru and 115°C for Rh in a glycerol bath, before being quenched. The obtained NPs were precipitated by acetone and redispersed in ethanol several times, before stabilizing them in water as the suspension medium.

### 2.3. Characterization Techniques

The crystal structures of the as-prepared materials in powder form, after drying the collected particles in a vacuum oven overnight, were investigated by X-ray powder diffraction (Panalytical Xpert Pro alpha powder, PANalytical) with Cu K*α* radiation (*λ* = 1.54056 Å). The dry particle size, morphology, and crystallinity of the samples were studied using transmission electron microscopy (TEM) (JEM-2100F, 200 kV, JEOL). The samples were prepared by drop casting ∼20 *µ*l of colloidal suspension on a TEM grid and allowing them to dry overnight. From the TEM micrographs, the size of the NPs was measured on at least 200 NPs/clusters in different fields of view. Dynamic light scattering (DLS, Malvern Nano-ZS90) was used to investigate the hydrodynamic size distribution of the as-prepared particles dispersed in DI water adjusted to pH 7.5. Inductively coupled plasma–optical emission spectroscopy (ICP-OES) (iCAP 6000 series, Thermo Scientific) had been used for the determination of the elemental composition of the as-synthesized materials prior to XRF phantom tests.

### 2.4. XRF Phantom Tests

In order to demonstrate the XRF contrasting properties of the synthesized NPs, experiments were performed using the laboratory XRF tomography system on a phantom of known dimensions [27]. The phantom consists of a 20 mm diameter cylinder made of soft-tissue equivalent material polyethylene terephthalate (PET). A central hole in the phantom with 2 mm diameter was filled with NP solution containing 1000 ppm of the XRF emitting elements (Y, Zr, Nb, Mo, Rh, and Ru), containing one probe element at a time. Incident on the phantom was an X-ray pencil beam with a ∼0.1 × 0.1 mm focus, created by multilayer elliptical optics focusing incoming X-rays from a high-brightness microfocus liquid-metal-jet source (D2, Excillum AB, Sweden). The beam was positioned so it penetrated the hole filled with NPs with a measured flux of ∼2.5 × 10^7^ ph/s. The edge of the phantom was 4 mm away from a photon-counting silicon drift detector (SDD) (Rayspec, United Kingdom) with an active area of 150 mm and energy resolution of ∼260 eV in the region of interest, placed at a 90° angle relative to the pencil beam.

The XRF spectra were recorded at the detector for each element. Additionally, the background signal was recorded using the same phantom filled with water. This background spectrum was subtracted from the XRF spectra to isolate the K-emission of the individual elements.

## 3. Results and Discussion

Due to the nature of the selected materials, different synthetic schemes have been applied for different material families. The ceramic materials containing Y, Zr, and Nb are synthesized by a hydrothermal method, using urea decomposition at elevated temperatures for the hydrolysis of the metal ions. Metallic nanostructures of Ru and Rh have been synthesized by a polyol reduction route. NPs have been evaluated for their crystal structure, composition, and morphology using various techniques.

### 3.1. X-Ray Powder Diffraction Analysis

**Figure 1:**
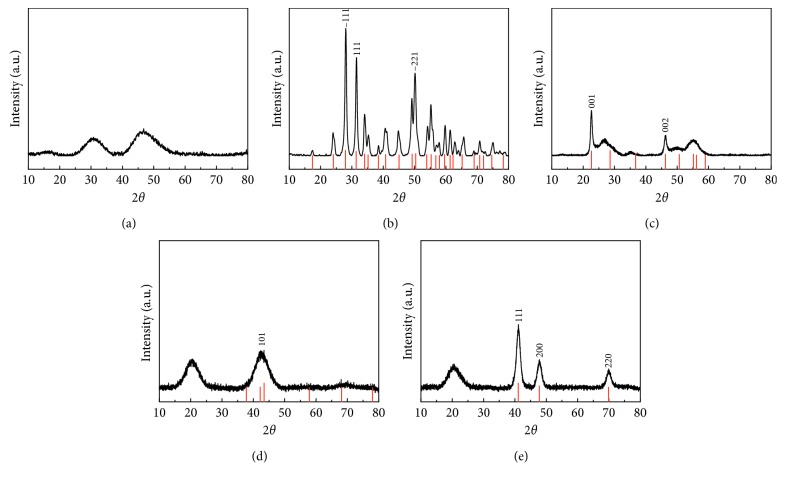
X-ray powder diffraction patterns of as-synthesized nanomaterials containing (a) yttrium, (b) zirconium, (c) niobium, (d) ruthenium, and (e) rhodium. Indexing of the diffraction peaks are performed for the corresponding phases as displayed by the reference patterns underneath the XRPD patterns, where index patterns correspond to (b) ZrO_2_ (ICDD card: 00-001-0750), (c) Nb_2_O_5_ (ICDD card: 00-028-0317), (d) Ru (ICDD card: 01-089-4903), and (e) Rh (ICDD card: 03-065-2866).

X-ray powder diffraction (XRPD) analyses were performed on all as-made nanopowders to identify the dominant crystalline phase. XRPD patterns are presented in Figures 1(a)–1(e). In Figures 1(a)–1(c) the XRPD patterns of the ceramic nanomaterials are presented, while Figures 1(d)–1(e) display the patterns of the metallic ones.

Nanopowders containing Y (Figure 1(a)) display two broad diffraction peaks at 30° and 45°, revealing the amorphous nature of the material, which has been confirmed to be Y(OH)CO_3_ on the basis of previous reports [29, 32]. As for the Zr-based material, the XRPD pattern (Figure 1(b)) shows a good match with ZrO_2_. All observed peaks can be indexed to the monoclinic ZrO_2_ phase (ICDD card: 00-001-0750), which is the low-temperature (<1100°C) phase [33, 34]. The most intense three peaks are marked with their corresponding Miller indices. The Nb-based material displays some clearly resolved diffraction peaks along with a series of broad peaks (Figure 1(c)). This indicates the copresence of partly crystalline and partly amorphous phases. The crystalline phase is identified as Nb_2_O_5_ and the corresponding diffraction peaks are indicated by the Miller indices, indexed for the orthorhombic phase of Nb_2_O_5_ (ICDD card 00-028-0317) [35]. Ru (Figure 1(d)) and Rh (Figure 1(e)) XRPD patterns show a good match with the corresponding metallic phases of Ru and Rh, respectively. The results agree well with the earlier reports using the same synthesis methods [20]. Nevertheless, there is a diffraction peak around 20° for both the products, which is attributed to PVP that forms a capping layer on the formed NPs [36] or the residual solvent ethylene glycol from the washing process.

### 3.2. Electron Microscopy Analysis

**Figure 2:**
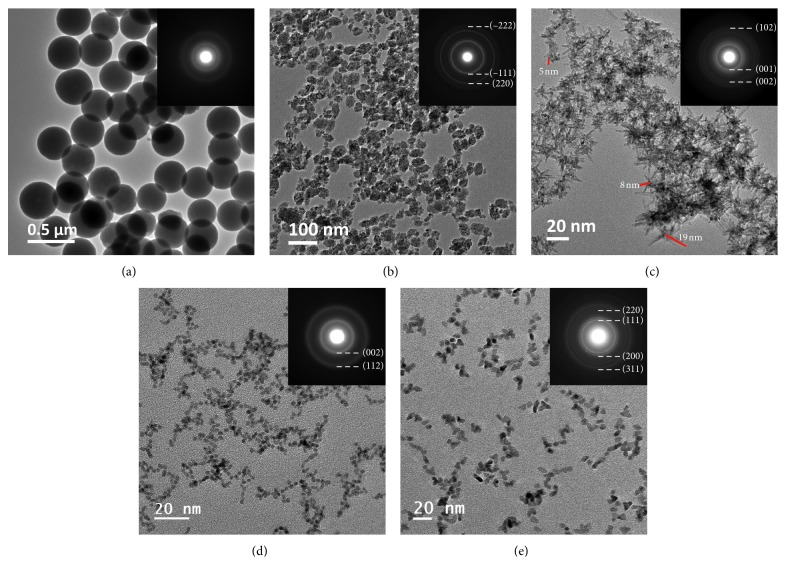
TEM micrographs of as-prepared particles of (a) Y(OH)CO_3_, (b) ZrO_2_, (c) Nb_2_O_5_, (d) Ru, and (e) Rh. Insets show the respective indexed SAED patterns of as-made NPs.

Morphology evaluation was performed on all as-made nanopowders, and representative TEM micrographs from each material group, along with indexed selected area electron diffraction (SAED) patterns, are presented in Figure 2. For all NPs, the SAED patterns confirmed the XRPD interpretations. Size-distribution histograms obtained from the TEM micrographs are presented in Figure S1.

The Y(OH)CO_3_ particles are presented in Figure 2(a), which display a perfect spherical morphology, with particle sizes ranging from 300 nm to 400 nm, with an average size of 350 nm. In the SAED pattern, the diffuse concentric rings demonstrate its amorphous nature, confirming the XRPD results. ZrO_2_ particles, presented in Figure 2(b), show irregular shapes and nonuniform sizes making it difficult to distinguish the discrete particles. Secondary clusters with sizes <50 nm are visible, where the primary particles observed are around 5–6 nm. The SAED pattern is indexed for the monoclinic ZrO_2_ phase (ICDD card: 00-001-0750), where the strong concentric rings reveal the polycrystalline nature of the material. Nb_2_O_5_ powders in Figure 2(c) exhibit agglomerated nanorod assemblies, where the nanorods' length is in the range of 8–20 nm with a cross section of ca. 5 nm. The diffuse SAED ring pattern, indexed for the orthorhombic Nb_2_O_5_ phase, reveals the amorphous character of the NPs. Ru particles, in Figure 2(d), show a spherical morphology and well-dispersed NP assembly. The particles exhibit a narrow size distribution with an average diameter of about 4 nm. SAED patterns reveal the amorphous character of the NPs, where the diffraction rings are indexed for metallic Ru, confirming XRPD results. Rh particles, in Figure 2(e), are also well-dispersed with a spheroid morphology, where the average NP size is around 8 nm. SAED patterns with strong concentric rings reveal the crystalline nature of the material.

Hydrodynamic size distribution of all the samples is evaluated by DLS, and the data are presented in Figure S2 and Table S1. For all the samples, the DLS size is larger than the dry particle size. This is mainly due to the solvation of the particles, the ligand capping around the NPs, or the clustering of NPs increasing their apparent hydrodynamic volume.

### 3.3. XRF Phantom Test

Our preliminary evaluation of the selected materials using Monte Carlo simulations [37] allowed us to evaluate the signal to noise ratio (SNR) for the expected K-emissions (see the Supplementary Material, section XRF Tomography and Table S2, Figures S3 and S4 for further details). In the phantom tests the XRF signal is proportional to the concentration of the emitting elements, regardless the composition, or size, of the NPs. Therefore, the metallic content in each NP system has been determined by ICP analysis in order to assure the presence of the same concentration (1000 ppm) of the element of interest in the phantom experiments. It is important to note that the percentage of the XRF active element in the synthesized NPs show variations based on the material compositions obtained. Rh and Ru are purely contrast-generating materials, while Y(OH)CO_3_ contains only 54% Y. For a given concentration of XRF active agent, the required weight of the material for a known volume can be twice as high for.

**Figure 3:**
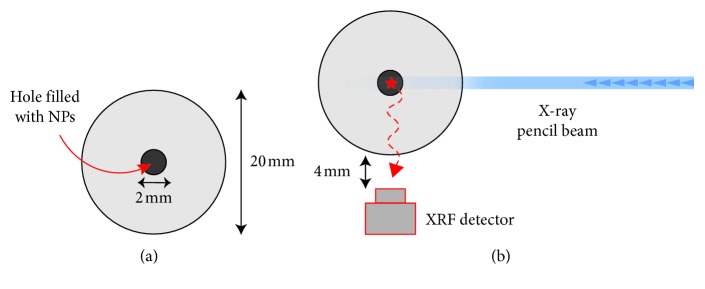
Experimental setup. (a) Cylindrical phantom made of PET with the central hole filled with NPs (b) Measurements of XRF by placing the phantom in the pencil beam and the detector at 90°.

Y in the form of Y(OH)CO_3_ as compared to Rh and Ru. In addition to the materials synthesized in this work, we have also included previously reported MoO_x_ NPs [28] in our phantom tests for comparison. The details of all prepared samples, their composition, and size distribution are summarized in Table 1. The experimental setup is described in Section 2.4 and schematically presented in Figure 3. For each set of NPs, the phantom was exposed to X-rays for 5 minutes, while the background measurement was performed for 30 minutes. The latter was recorded by simply filling the phantom with water and recording the background scattering. The reason for the longer background measurement was to reduce errors when subtracting it from the respective XRF spectra (thus separating the signals from the background). It should be noted, however, that these long exposure times are used only to qualitatively demonstrate the XRF properties of these NPs. In a real tomographic setting, much shorter exposure times are necessary.

**Figure 4:**
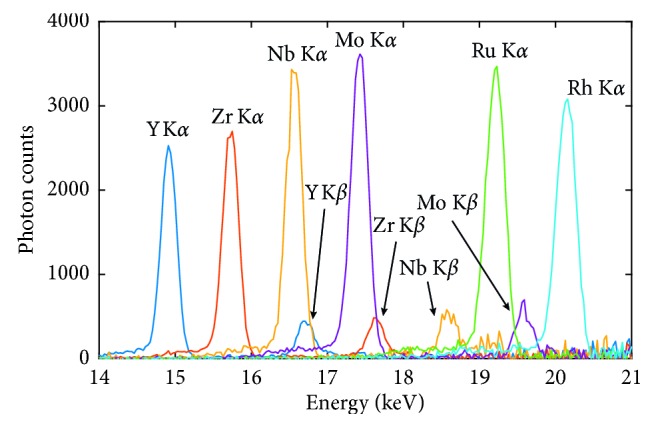
Overlay of XRF spectra separately recorded for the different nanoparticles, with the background subtracted.

Figure 4 shows a comparison of the XRF signal for the different NPs containing 1000 ppm of the XRF-emitting elements (Y, Zr, Nb, Mo, Rh, and Ru), based on the analysis of six sample solutions containing one probe element at a time. The K*α* emission for these elements together with a narrow pencil beam can be used to probe the spatial concentration of NPs inside a soft-tissue equivalent object. The background contribution is considered low within the energy window of 14–19 keV, with K*β* also visible for most elements. There is a trade-off between energy matching, fluorescence yield, and detection efficiency. For the higher-Z elements such as Mo, Ru, and Rh, the K-absorption edges (20, 22.1, and 23.2 keV, respectively) lie closer to the excitation energy of the source (24 keV). This results in a larger probability of photoelectric absorption and hence, higher fluorescence yield. However, since the absorbing element of the detector (SDD) is made out of silicon, detection probability is lower for higher energy K*α* emission. For the lower-Z elements, Y, Zr, and Nb experience lower fluorescence yield. This is due to larger energy mismatch as well as higher self-absorption inside the phantom, owing to the lower K*α* energy although the absorption efficiency of the SDD is higher at these energies. It should also be noted that, while not apparent in the figure, there is a background contribution present from multiple Compton scattering of the excitation beam inside the soft-tissue equivalent phantom. By the nature of Compton scattering, the background contribution is higher toward the higher energy end of the spectrum in Figure 3. The XRF phantom test results show that Mo and Nb give optimum signal levels with low background levels and good SNR. Although Mo and Nb show promising XRF performance, Y, Zr, Rh, and Ru elements offer an alternative platform for the ongoing work on the investigation of the biocompatibility of the studied library of materials. Ultimately, the biocompatibility of these elements, and their corresponding compositions, is the most crucial aspect that will dictate their possible use and dosage in a biologically relevant context.

## 4. Conclusions

We successfully fabricated a library of NPs, selected based on their K-emission lines, as potential XRF contrast agents for X-ray tomography bioimaging in the small-animal energy regime. The composition and morphology of the as-synthesized NPs were evaluated using XRPD and TEM techniques. Materials showed different degrees of crystallinity with the corresponding crystal structure and composition identified as hydroxy carbonate for Y, oxide for Zr and Nb, and the metallic phase for Ru and Rh, revealing the success of the synthetic processes used. The dominant morphology was spherical/spheroid for almost all NP systems, except Nb where mainly nanorod morphology was observed. Phantom tests were performed using our laboratory XRF tomography system, where K-emission lines have been recorded successfully, well separated from the background. Our results demonstrate the promise of these NPs as XRF contrast agents for small-animal laboratory XRF tomography. Furthermore, the broad variation in the materials' composition offers a rather flexible platform for tailoring their design parameters as morphology, size, crystallinity, and surface chemistry. The fine adjustment of these characteristics will lead to their improved stability in biological media along with none or low toxicity. These are important requirements for tuning the applied dose of the nano probes, translating their use in in vivo XRF tomography.

## Figures and Tables

**Table 1:** Details on the characteristics of the investigated nanomaterials.

Element	Compound	Synthetic route	Avg. particle size (nm)	Remarks
Y	Y(OH)CO_3_	Hydrothermal	350	Spherical
Zr	ZrO_2_	Hydrothermal	<50	Clusters
Nb	Nb_2_O_5_	Hydrothermal	<20	Nanorods
Mo	MoOx^29^	Hydrothermal	10 nm	Clusters
Ru	Ru	Reduction in solution	4	Spherical
Rh	Rh	Reduction in solution	8	Polygonal

## Data Availability

No data were used to support this study.

## Supplementary Materials

Supplementary Materials Supplemental Figure 1: size distribution histograms of as-made NPs obtained from TEM micrographs. Supplemental Figure 2: hydrodynamic size distribution of NPs as obtained from DLS measurements. Supplemental Figure 3: the setup used for Monte Carlo simulation geometry. Supplemental Figure 4: simulated XRF signals from the selected elements. Supplemental Table 1: TEM and DLS size distribution and polydispersity index of as-prepared NPs. Supplemental Table 2: SNR and SBR ratio from simulation of XRF signals relative to Mo.
