# Supplementary material for: A Library of Potential Nanoparticle Contrast Agents for X-Ray Fluorescence Tomography Bioimaging
Source: Contrast Media Mol Imaging. 2018 Dec 27;2018:8174820. doi: 10.1155/2018/8174820 (PMC6327276; doi:10.1155/2018/8174820)
Supplement: Supplementary Materials — Supplemental Figure 1: size distribution histograms of as-made NPs obtained from TEM micrographs. Supplemental Figure 2: hydrodynamic size distribution of NPs as obtained from DLS measurements. Supplemental Figure 3: the setup used for Monte Carlo simulation geometry. Supplemental Figure 4: simulated XRF signals from the selected elements. Supplemental Table 1: TEM and DLS size distribution and polydispersity index of as-prepared NPs. Supplemental Table 2: SNR and SBR ratio from simulation of XRF signals relative to Mo. [file 8174820.f1.pdf]

Supplementary Materials

A library of potential nanoparticle contrast agents for X-ray fluorescence tomography bio-imaging

Yuyang Li[1], Kian Shaker[1], Jakob C. Larsson[1], Carmen Vogt[1], Hans M. Hertz[1], Muhammet S. Toprak[1]

[1]Department of Applied Physics, Biomedical and X-ray Physics, KTH Royal Institute of Technology/AlbaNova, SE 106 91 Stockholm, Sweden.

Corresponding author: toprak@kth.se

Particle Size Analysis by TEM and DLS

Particle size for the as-made NPs were estimated from TEM micrographs, by counting at least 200 NPs, or clusters in case if ZrO_2. Corresponding histograms are presented in Figure S1 for Y, Zr, Ru and Rh based materials with dominantly spherical/spheroid morphology, while it is omitted for Nb material which display highly anisotropic nanorod morphology. Table S1 summarizes the results, where average size of dry NPs obtained are 350 nm for $Y(OH)CO_3$, 45 nm for ZrO_2, 8 nm for Ru and 3 nm for Rh.

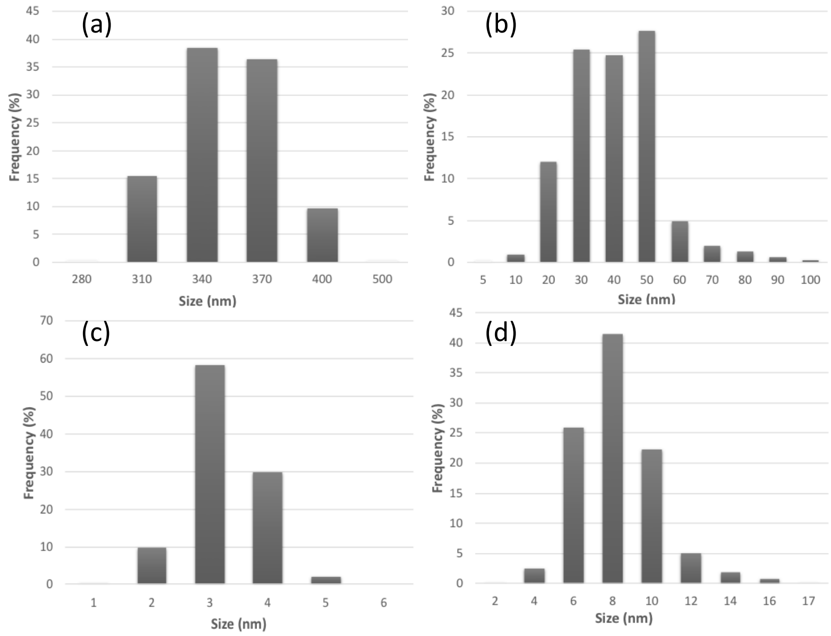

Figure S1. Histograms obtained from the TEM images, representing size distribution of as-prepared particles after drying for (a) $Y(OH)CO_3$, (b) ZrO_2, (c) Ru, and (d) Rh.

The DLS analysis results are presented in Figure S2 and are summarized in Table S1. Results revealed the fact that the hydrodynamic diameter of the synthesized NPs in DI water is slightly larger than the size of the NPs estimated from TEM. It is generally expected that the hydrodynamic particle size estimated from DLS is larger than the dry size of particles estimated from TEM due to the solvation layer, the ligand capping around the NPs, or clustering of NPs, increasing their apparent hydrodynamic size. Polydispersity index (PDI) can be used to assess the distribution of the particle size, and it ranges between 0 and 1. A small PDI reveals a narrow size distribution. Obtained PDI values for these NPs range between 0.15 and 0.30, except for Nd sample with PDI of 0.54, which may indicate that although there is clustering of NPs, the width of the distribution is not broad. The effective size distribution can be significantly influenced by different dispersion scenarios, or coating the NPs with more charge bearing inorganic or organic moieties. This has not been investigated in detail as it was out of the scope of this work.

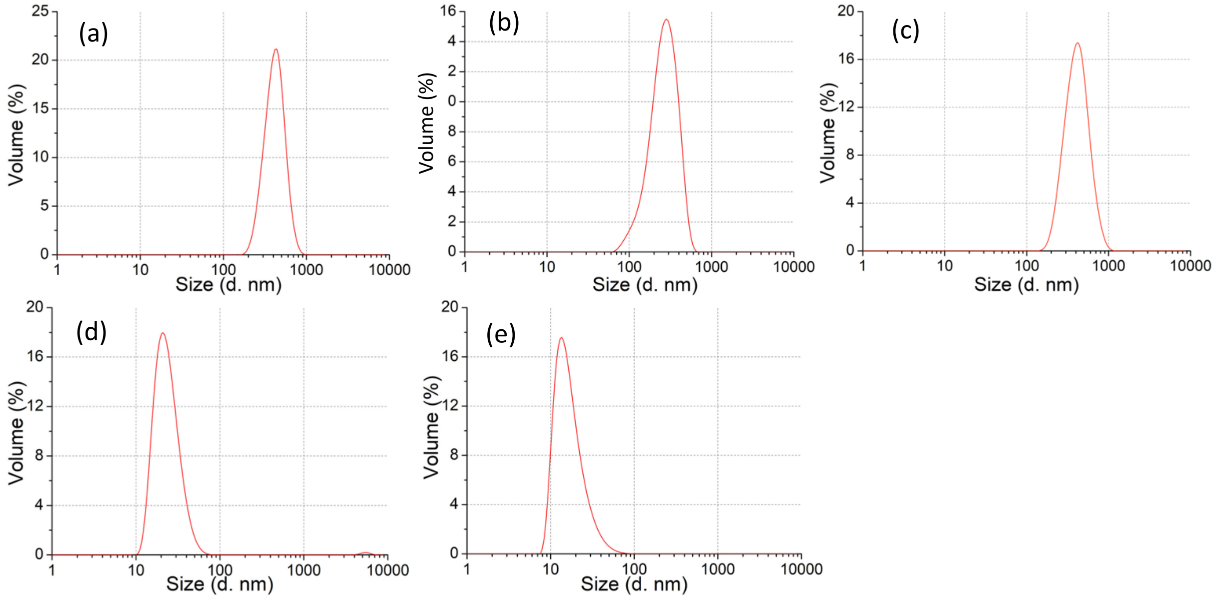

Figure S2. DLS size distribution of as-prepared particles of (a) $Y(OH)CO_3$, (b) ZrO_2, (c) Nb_2O_5, (d) Ru and (e) Rh.

Table S1. TEM and DLS size distribution and polydispersity index of as-prepared NPs.

Element	Compound	TEM Average Size [nm]	DLS Average Size [nm]	PDI (DLS)
Y	$YOH(CO_3)$	350	375	0.15
Zr	ZrO_2	40	275	0.18
Nb	Nb_2O_5	--	420	0.54
Ru	Ru	4	25	0.25
Rh	Rh	8	17.5	0.30

XRF Simulation

Simulation setup

The software PENELOPE2011 was used for all Monte Carlo simulations.

The simulation was set up to be as close to the real setup as possible. The worst case scenario, where the detector is 18.5 mm away from the center of the object was simulated. A 120 second long exposure using a 100 mm^2 detector was used to get sufficient statistics. The simulation geometry is presented in Figure S3. Specifications of the set-up are as follows:

- The object is a 2 cm diam. cylinder of soft tissue.
- The NPs were simulated as a 500 μm diam. cylinder in the center of the object. The particles had a concentration of 0.1% by weight of soft tissue.
- The detector was placed 18.5 mm from the center.

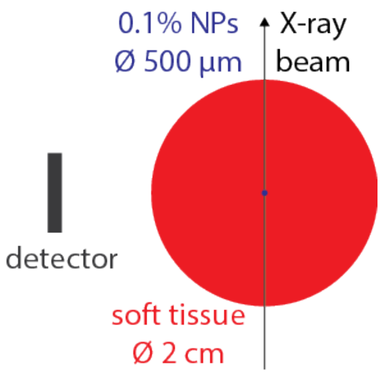

Figure S3. The set-up used for Monte Carlo simulation geometry.

Signal-to-Noise (SNR) and Signal-to Background Ratio (SBR) calculation

Photons follow Poisson statistics where

$$signal = N_{photons}\ ,\ variance = N_{photons},$$

so

$$\mathrm{SNR} = \frac{signal}{\sqrt{variance}} = \frac{N_{flourescence}}{\sqrt{N_{fluorescence} + N_{Compton}}}\ ,$$

and

$$\mathrm{SBR} = \frac{N_{flourescence}}{N_{Compton}}\ .$$

Table S2 shows SNR and SBR ratios for the different elements relative to Mo, where the signals were integrated over the FWHM of the Kα peak.

Table S2. SNR and SBR ratio from simulation of XRF signals relative to Mo.

	39 Y	40 Zr	41 Nb	42 Mo	44 Ru	45 Rh
$\overline{K_{\alpha}}$ (keV)	14.9	15.7	16.6	17.4	19.2	20.2
SNR (Ø 2 cm)	-20%	-11%	-6%		-10%	-31%
SBR (Ø 2 cm)	200	30	5	1	0.04	0.01

Obtained XRF signals are plotted in Figure S4.

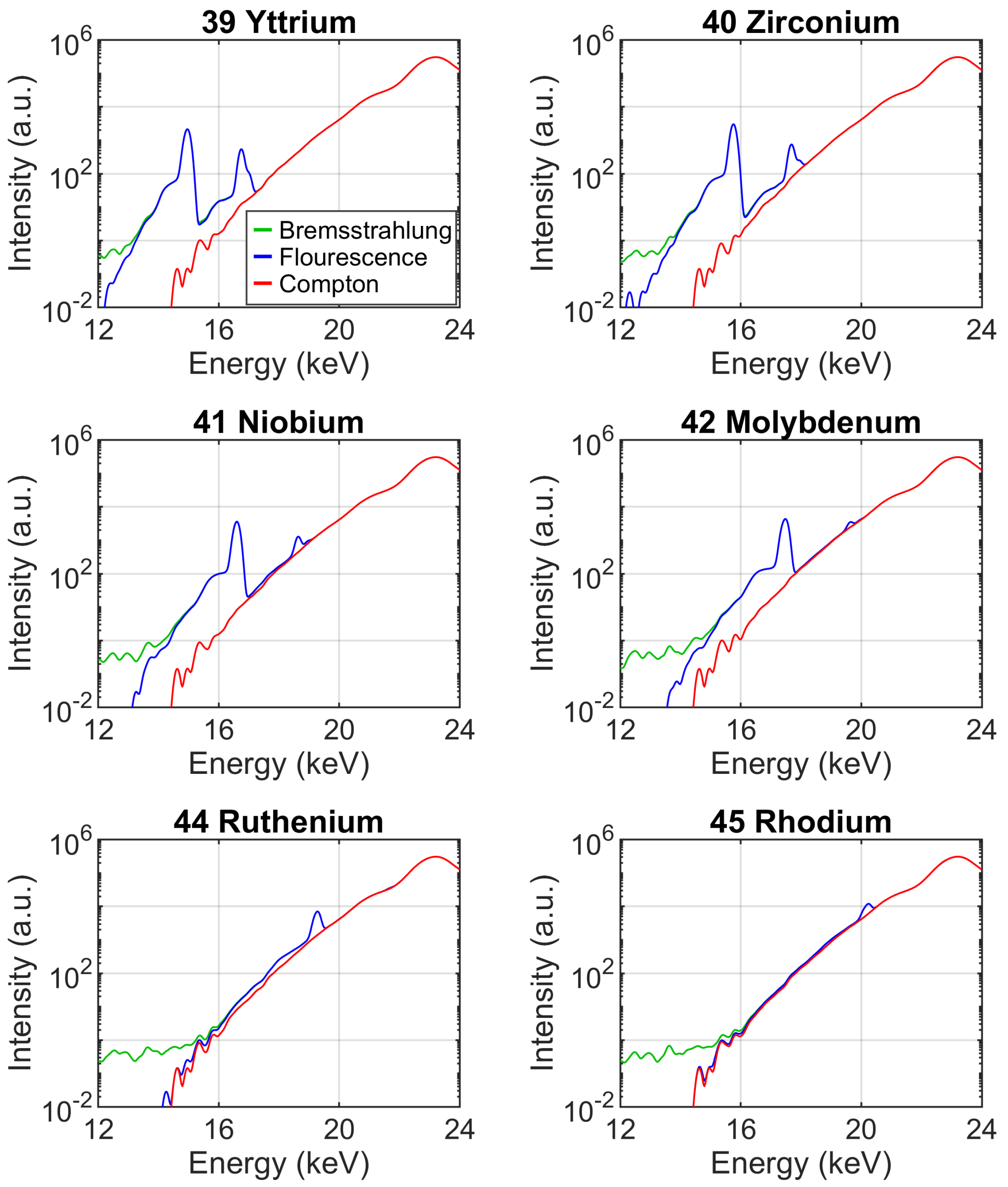

Figure S4. Simulated XRF signals from the selected elements using the simulation geometry presented in Figure S3.
